# The Usefulness of a Smartphone App–Based Smoking Cessation Program for Conventional Cigarette Users, Heated Tobacco Product Users, and Dual Users: Retrospective Study

**DOI:** 10.2196/42776

**Published:** 2023-03-17

**Authors:** Yuko Noda, Ryuhei So, Misaki Sonoda, Takahiro Tabuchi, Akihiro Nomura

**Affiliations:** 1 Department of Biomedical Informatics CureApp Institute Karuizawa Japan; 2 Department of Transdisciplinary Sciences for Innovation Kanazawa University Kanazawa Japan; 3 CureApp Inc Tokyo Japan; 4 Cancer Control Center Osaka International Cancer Institute Osaka Japan; 5 Tokyo Foundation for Policy Research Toyko Japan; 6 Innovative Clinical Research Center Kanazawa University Kanazawa Japan; 7 Department of Cardiovascular Medicine Kanazawa University Graduate School of Medical Sciences Kanazawa Japan

**Keywords:** smoking cessation, nicotine dependence, digital therapeutics, telemedicine, telecare, mobile phone, smoking cessation program, online counseling, online therapy, heated tobacco product, HTP

## Abstract

**Background:**

Heated tobacco products (HTPs) are widespread in Japan, and smoking cessation of such products has become an important issue owing to the spread of harmful effects from HTPs. The efficacy of online digital therapy has been reported in smoking cessation treatment; however, we have limited evidence of online smoking cessation programs for HTP users.

**Objective:**

In this study, we evaluate the usefulness of the Ascure program for HTP users (defined as exclusive HTP use or dual use of HTP and cigarettes) compared with exclusive cigarette users.

**Methods:**

This was a retrospective study. We recruited adult smokers participating in the Ascure online smoking cessation program in Japan from June 2019 to February 2021. The Ascure smartphone app provided four elements: (1) educational video tutorials to enhance the understanding of nicotine dependence, (2) a personalized to-do list for behavior change, (3) a digital diary for record keeping, and (4) interactive chat sessions for relief from cravings or withdrawal symptoms. The primary outcome was the continuous abstinence rate (CAR) at weeks 21 to 24, biochemically validated using salivary cotinine testing. We considered those who dropped out of the program as smoking cessation failures. We analyzed the primary outcome using inverse probability weighting against tobacco product type estimated by multinomial propensity scores. We also assessed CAR at weeks 9 to 12 and program adherence.

**Results:**

We analyzed data from 2952 participants, including 52% (1524/3478) in the cigarette group, 35% (1038/3478) in the HTP group, and 13% (390/3478) in the dual-use group, who had a mean age of 43.4 (SD 10.8) years and included 17% (513/2952) women. CAR at weeks 21 to 24 showed that exclusive HTP users were more likely to stop tobacco use than exclusive cigarette smokers (CAR 52.6% for cigarette users vs CAR 64.8% for HTP users; odds ratio [OR] 1.17, 95% CI 1.12-1.22; *P*<.001). There was no significant difference between the exclusive cigarette users and the dual users (CAR 52.6% for cigarette users vs CAR 48.7% for dual users; OR 0.99, 95% CI 0.93-1.05; *P*=.77). CAR at weeks 9 to 12 was 56.7% (95% CI 54.2%-59.2%) for the exclusive cigarette users, 68.3% (95% CI 65.5%-71.1%) for the exclusive HTP users, and 58.2% (95% CI 53.3%-63.1%) for the dual users. The program adherence rate at week 24 was 70.7% overall (68.4% for cigarette users, 75% for HTP users, and 67.9% for dual users).

**Conclusions:**

Exclusive HTP users had higher CARs and adherence compared with exclusive cigarette users, indicating a higher affinity for the Ascure online smoking cessation program. This program might be a useful smoking cessation option for HTP users, as well as for cigarette smokers.

## Introduction

Smoking causes many diseases, most notably cardiovascular disease, chronic obstructive pulmonary disease, and cancer. Smoking is one of the major risk factors for adult mortality from noncommunicable diseases in Japan [1,2]. Despite the government’s smoking cessation efforts, such as increasing tobacco taxes and promoting the prevention of passive smoking, the national adult smoking rate remains high in Japan as of 2019: 27.1% for men and 7.6% for women [3,4]. Tobacco companies have also aggressively promoted heated tobacco as an alternative product to conventional cigarettes. Since Philip Morris launched the world’s first heated tobacco product (HTP)—iQOS—in Japan and Italy in 2014, 27.2% of male and 25.2% of female smokers in Japan now use the product. [4] In addition, 6.9% of men and 4.8% of women reported dual use of conventional cigarettes and HTPs [4]. Since HTPs have similar hazardous effects, such as cardiovascular disease, as traditional cigarettes [5,6], it is necessary to encourage people to quit using both HTPs and conventional cigarettes [7,8].

A standard smoking cessation program is provided at outpatient clinics in Japan for people with nicotine dependence. Moreover, HTP users have been eligible for smoking cessation treatment under general health insurance since 2020. The standard smoking cessation program consists of pharmacotherapy using varenicline or a nicotine patch and physician counseling for 12 weeks in-person or through telemedicine [9]. However, the program’s completion rate is low (36%), because most patients receiving smoking cessation treatment are of working age and are too busy to visit outpatient clinics [10]. In addition, the continuous abstinence rate (CAR) drops significantly after completing the 12-week program [11].

To reduce program withdrawal and to support long-term smoking cessation, CureApp, Inc released the Ascure online smoking cessation program [12-14]. The program provides complete face-to-face telemedicine services using pharmacotherapy for physical dependence and behavioral therapy for psychological dependence. Interventions that combine pharmacotherapy and behavioral therapy increase smoking cessation success [15,16]. We previously reported that the smoking cessation success rate of the Ascure program was favorable among exclusive cigarette users. However, there is no evidence of usefulness regarding the Ascure program for HTP users. Hence, we performed a retrospective analysis to evaluate the usefulness of the Ascure program for people using HTPs, including exclusive HTP users and dual users of HTPs and cigarettes, compared with exclusive cigarette users.

## Methods

### Study Design

This was a retrospective study that evaluated the usefulness of the Ascure online smoking cessation program for HTP users. In brief, we divided the program’s participants into 3 groups based on their tobacco product use: exclusive cigarette users, exclusive HTP users, and dual users of cigarettes and HTPs. Then, we compared the smoking cessation success rate of the exclusive cigarette group (as a reference) with the exclusive HTP and dual-use groups. The primary endpoint was the biochemically validated CAR at weeks 21 to 24 (CAR 21-24). This study followed the Strengthening the Reporting of Observational Studies in Epidemiology (STROBE) checklist.

### Ethical Considerations

This study was conducted in accordance with the Declaration of Helsinki, the Ethical Guidelines for Medical and Biological Research Involving Human Subjects, and all other applicable laws and guidelines in Japan. The study protocol was approved by the Institutional Review Board of Kanazawa University; the study approval number was 2021-184 (113839). Study data were anonymized, so no written consent was obtained. However, prior to using the Ascure smartphone app, participants were clearly informed that the app’s data would be used for research; only those who consented to this could use the app.

### Participants

We recruited adult smokers who participated in the Ascure online smoking cessation program in Japan from June 2019 to February 2021. We included participants who met all of the following criteria: (1) they were enrolled in the affiliated Japanese health insurance association, (2) they were willing to quit using tobacco immediately, (3) they could use a smartphone (Android 5.0 or higher or iOS 10.0 or higher), and (4) they agreed to participate in the smoking cessation program in the app. We excluded participants who had severe mental illness or difficulty continuing the entire program. For analysis, we also excluded participants without sufficient baseline information.

### Outcomes

The primary outcome was CAR 21-24. We defined smoking cessation success as self-reported successful smoking cessation for the past month during an interview and a confirmed negative result in salivary cotinine testing [17]. We performed salivary cotinine testing using the iScreen cotinine oral fluid screening device (Abbott Diagnostics Medical Co). We considered those who dropped out of the program as smoking cessation failures. We used the Nicotine Dependence Cognition Scale (NDCS), which indicates the severity of nicotine dependence and cognitive impairment in smokers (Multimedia Appendix 1) [14,18]. The secondary outcomes were CAR at weeks 9 to 12 (CAR 9-12), the impact of tobacco products on the progress of quitting smoking, and program adherence rates.

### Ascure Online Smoking Cessation Program

**Figure 1:**
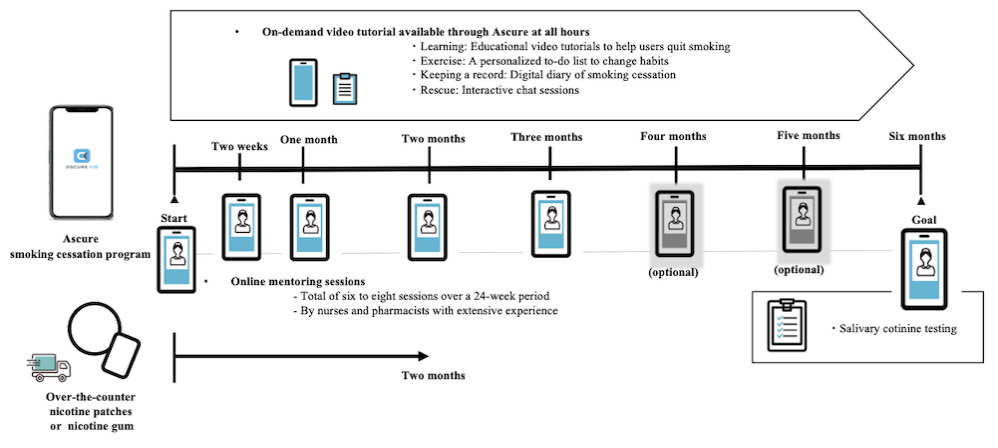
Overview of the Ascure online smoking cessation program.

Figure 1 provides an overview of the Ascure online smoking cessation program. Participants can receive the on-demand video tutorial through the app anywhere and at any time; there are 6 to 8 online-mentoring smoking cessation counseling sessions conducted by experienced nurses and pharmacists. All online counselors completed in-house training before conducting the interviews; the training included instructions on smoking cessation as well as skills on how to explain the appropriate use of the app. In addition, the participants could concurrently use over-the-counter nicotine patches or nicotine gum for 8 weeks.

The app consists of four elements: (1) learning (educational video tutorials to help users quit using tobacco), (2) exercise (a personalized to-do list to change habits), (3) keeping a record (a digital diary of smoking cessation), and (4) rescue (interactive chat sessions for relief from cravings or withdrawal symptoms). CAR was assessed among the participants with salivary cotinine testing at 6 months.

The Ascure smartphone app was released by CureApp, Inc. Participants can download the app to their own smartphones and start using it by entering a unique passcode individually issued by CureApp. After installing the app, participants proceed to an appointment for the first interview if they agree to the personal information protection regulations, including consent for the anonymized information data analysis. Details of the app have been provided elsewhere [17].

### Data Collection

We collected each participant’s information, including use of tobacco products by type, through the app and counseling sessions. The counselors determined the success or failure of smoking cessation at each interview based on participants’ self-reports. We also collected results from the salivary cotinine testing. The testing device was delivered to the participants’ homes and used by them under supervision during the final interview. The counselor visually confirmed and collected the test results (positive or negative). Data acquired by the app included age, sex, number of cigarettes smoked per day, years of tobacco use, and motivation to quit using tobacco.

### Statistical Analysis

Baseline characteristics are described as the mean (SD) for continuous variables and number with percentage for categorical variables. We analyzed the primary outcome using inverse probability weighting (IPW) against tobacco product type estimated by multinomial propensity scores based on 5000 regression trees and the average treatment effect on the treated. The propensity model included age, sex, Brinkman index, cigarettes smoked per day, years of tobacco use, number of smoking cessation attempts before the study, and NDCS score as covariates [19,20]. We present maximum standardized differences before and after IPW for each baseline characteristic. We defined a maximum standardized difference of 0.2 or higher as indicative of imbalance [19,20]. All analyses were performed using R (version 3.6.3; R Foundation for Statistical Computing), with *P* values <.05 deemed significant.

## Results

### Baseline Characteristics

**Figure 2:**
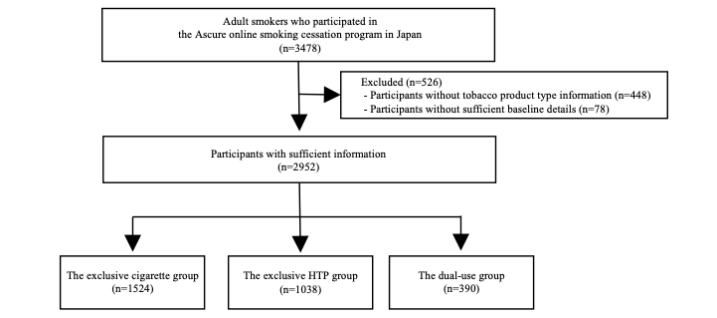
Study flowchart. HTP: heated tobacco product.

Figure 2 shows the study flowchart. A total of 3478 individuals participated in the Ascure online smoking cessation program. Of these, we excluded 448 participants without tobacco product type information and 78 without sufficient baseline details. Thus, we enrolled 2952 participants (1524 for the exclusive cigarette group, 1038 for the exclusive HTP group, and 390 for the dual-use group) for further analysis.

Table 1 shows the participants’ characteristics at baseline before IPW. The mean age was 43.4 (SD 10.8) years, 17% (513/2952) of participants were women, and the mean duration of tobacco use was 22 years. We used IPW to minimize differences in baseline characteristics between the groups. The characteristics were well-balanced after applying IPW: all maximum standardized differences among the groups were ≤.05 (Table 1).

**Table 1:** Participants’ clinical characteristics at baseline.

	Total (n=2952)	Exclusive cigarette users (n=1524)	Exclusive HTP^a^ users (n=1038)	Dual users (n=390)	Maximum standardized difference	
					Before IPW^b^	After IPW
Age (years), mean (SD)	43.4 (10.8)	44.6 (11.0)	42.2 (10.1)	41.4 (11.0)	0.30	0.05
Female, n (%)	513 (17.4)	286 (18.8)	178 (17.1)	49 (12.6)	0.16	0.05
Brinkman index, mean (SD)	392 (290)	411 (298)	376 (285)	364 (261)	0.16	0.00
Cigarettes per day, mean (SD)	17.5 (8.9)	17.2 (7.4)	17.9 (11.2)	17.4 (6.7)	0.10	0.04
Duration of tobacco use (years), mean (SD)	21.6 (10.8)	22.8 (11.2)	20.4 (10.0)	20.1 (11.0)	0.24	0.01
Number of smoking cessation attempts before the study, mean (SD)	1.7 (2.1)	1.7 (2.2)	1.6 (1.9)	1.6 (2.0)	0.07	0.05
NDCS^c^, mean (SD)	11.8 (3.5)	11.9 (3.6)	11.7 (3.5)	11.5 (3.5)	0.10	0.02

^a^HTP: heated tobacco product.

^b^IPW: inverse probability weighting.

^c^NDCS: Nicotine Dependence Cognition Scale.

### Overall Smoking Cessation Success Rate Using the Ascure Online Smoking Cessation Program

First, we evaluated the overall success rates of the Ascure online smoking cessation program. The overall biochemically validated CAR 9-12 was 61% (95% CI 59.2%-62.8%) and CAR 21-24 was 56.4% (95% CI 54.6%-58.2%).

### Primary Endpoint

**Figure 3:**
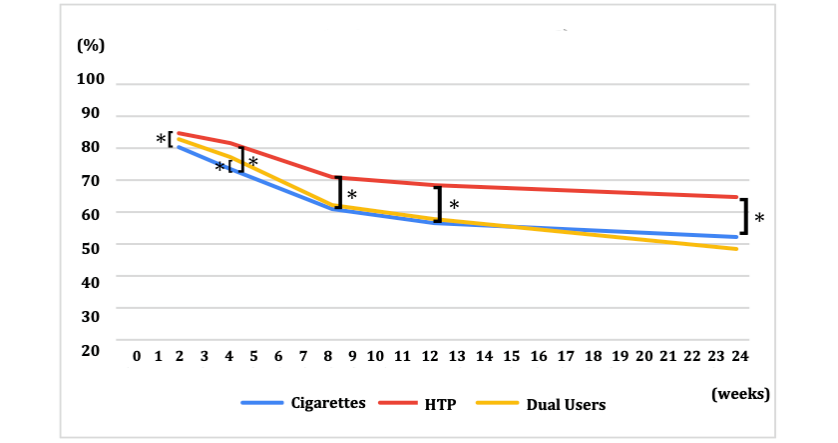
Continuous abstinence rate in the 3 groups. HTP: heated tobacco product. *P<.05 compared to exclusive cigarette users.

Figure 3 and Table 2 show the CARs in the 3 groups. The biochemically validated CAR 21-24 was 52.6% (95% CI 50.1%-55.1%) for the exclusive cigarette group, 64.8% (95% CI 61.9%-67.7%) for the exclusive HTP group, and 48.7% (95% CI 43.7%-53.7%) for the dual-use group. Compared with the exclusive cigarette group, the exclusive HTP group had significantly higher CAR 21-24 (OR 1.17, 95% CI 1.12-1.22; *P*<.001), whereas there was no significant difference between the exclusive cigarette and the dual-use groups (OR 0.99, 95% CI 0.93-1.05; *P*=.77).

**Table 2:** Continuous abstinence rates by tobacco products. *P* values were calculated using an inverse probability-weighted data set.

Time point	Continuous abstinence rate, % (95% CI)				HTP^a^ users vs cigarette users			Dual users vs cigarette users	
	Exclusive cigarette users	Exclusive HTP users	Dual users	Odds ratio (95% CI)		*P* value	Odds ratio (95% CI)		*P* value
Weeks 9 to 12	56.7 (54.2-59.2)	68.3 (65.5-71.1)	58.2 (53.3-63.1)	1.15 (1.10-1.19)		<.001	1.05 (1.00-1.12)		.07
Weeks 21 to 24	52.6 (50.1-55.1)	64.8 (61.9-67.7)	48.7 (43.7-53.7)	1.17 (1.12-1.22)		<.001	0.99 (0.93-1.05)		.77

^a^HTP: heated tobacco product.

### Secondary Endpoint

CAR 9-12 was 56.7% (95% CI 54.2%-59.2%) in the exclusive cigarette group, 68.3% (95% CI 65.5%-71.1%) in the exclusive HTP group, and 58.2% (95% CI 53.3%-63.1%) in the dual-use group. Compared with the exclusive cigarette group, the exclusive HTP group also showed significantly higher CAR 9-12 (OR 1.15, 95% CI 1.10-1.19; *P*<.001; Table 2). In contrast, there was again no significant difference between the exclusive cigarette group and the dual-use group (OR 1.05, 95% CI 1.00-1.12; *P*=.07).

Table 3 shows the proportions of success, failure, and drop outs for smoking cessation in the exclusive cigarette, exclusive HTP, and dual-use groups by week. The program adherence rate at week 24 was 70.7% overall, 68.4% in the exclusive cigarette group, 75% in the exclusive HTP group, and 67.9% in the dual-use group. CAR 21-24 among people who completed the program was 76.8% in the exclusive cigarette group, 86.4% in the exclusive HTP group, and 71.7% in the dual-use group.

**Table 3:** Continuous abstinence rates for smoking cessation among exclusive cigarette users, exclusive heated tobacco product users, and dual users, grouped by success, failure, and drop-out status and by week.

Groups		Continuous abstinence rate, %				
		Weeks 0-2	Weeks 3-4	Weeks 5-8	Weeks 9-12	Weeks 21-24
**Exclusive cigarette users**						
	Success	80.5	73.3	60.8	56.7	52.6
	Failure	14.4	15.9	21.9	20.1	15.9
	Dropout	5.1	10.8	17.3	23.2	31.6
**Exclusive heated tobacco product users**						
	Success	84.8	81.5	71.3	68.3	64.8
	Failure	11.1	9.9	13.5	12.2	10.2
	Dropout	4.1	8.6	15.2	19.5	25
**Dual users**						
	Success	83.1	77.2	62.1	58.2	48.7
	Failure	11.8	11.8	20.3	19	19.2
	Dropout	5.1	11	17.7	22.8	32.1

## Discussion

### Principal Findings

In this study, we evaluated the usefulness of the Ascure online smoking cessation program for people using HTPs compared to exclusive cigarette users. We found that there was a significantly higher CAR 9-12 and CAR 21-24 in the exclusive HTP group compared to the exclusive cigarette group, whereas there was no significant difference in CAR 9-12 or CAR 21-24 between the exclusive cigarette group and the dual-use group. In addition, the program adherence rates were favorable in all groups. Specifically, the HTP users had a higher adherence rate compared with the exclusive cigarette users.

### Comparison to Prior Work

This study had several findings. First, the biochemically validated CAR 9-12 and CAR 21-24 values were significantly higher in the exclusive HTP group compared to the exclusive cigarette group. There are several possible reasons for the favorable result. First, many people might use HTPs as assistance for smoking cessation [21], and these individuals tend to be highly motivated to quit using tobacco. Second, previous reports have shown that more HTP users than cigarette users have low nicotine dependence, which is a predictor of smoking cessation success, as well as having patient background conditions that are also predictors of success [19,22]. Moreover, because HTPs were not available prior to their launch in 2014, the exclusive HTP users had experienced a successful complete switch from conventional cigarettes to HTPs at least once during their average of 20 years of tobacco use. Past success in quitting smoking contributes to future success in quitting smoking [22]. Furthermore, a higher program adherence rate could have contributed to the increase in CAR. [9,17] The higher adherence rate among the exclusive HTP users compared to the exclusive cigarette users might be attributable to the favorable CAR. Nomura et al [23] previously reported the efficacy of an 8-week telemedicine smoking cessation program provided by primary physicians to nicotine-dependent people who used HTPs; the study had a 10-month follow-up period (sending surveys and smoking cessation advice via the app). They found that exclusive HTP users had significantly higher CAR 9-24 than exclusive cigarette users (53.8% for cigarette users vs 67% for HTP users). These reports are consistent with the present results, and they suggest the efficacy of online smoking cessation treatments for exclusive HTP users. In contrast, Kanai et al [24] reported that HTP users were less likely than exclusive cigarette users to quit tobacco in a prospective study. The HTP group in their study included both exclusive HTP users and dual users, which may have greatly influenced the results; it has been reported that the success rate for smoking cessation is lower among dual users [23]. In addition, a considerable difference in success rates between the exclusive cigarette groups in the 2 studies makes direct comparisons difficult and suggests that there were large differences in the epidemiological backgrounds of the participants or in the effects of the treatment. Therefore, it is important to interpret and compare the results while considering tobacco product use conditions. We report preferable smoking cessation success rates only using the Ascure program among exclusive HTP users.

Second, the biochemically validated CAR 9-12 and CAR 21-24 results were not significantly different in the dual-use group and the exclusive cigarette group in this study. Dual users could have advantages and disadvantages for smoking cessation. Advantages include the use of HTPs, which can assist with smoking cessation [21]. Moreover, dual users are already in the process of trying to cease smoking; they are also more likely to be male than conventional cigarette users, which is a predictor of successful smoking cessation [22]. Disadvantages of dual use include a tendency to feel inadequate because of not being able to use HTPs exclusively [25], which might inhibit smoking cessation success and a full transition to HTPs. Prior studies have reported lower smoking cessation rates for dual users as compared to exclusive cigarette users [23]. Another report showed no difference in smoking cessation behavior between dual users and cigarette users [26]. Thus, the present results might be the consequence of a balance between advantages and disadvantages for dual users. Other factors, such as lower age and lower recognition of the harms of smoking, are known to increase resmoking rates [27]. Dual users tend to have a larger CAR drop from week 12 to 24 than exclusive cigarette users, another risk factor for resmoking [26]. Thus, continued observation after the therapeutic intervention is particularly needed for dual users, considering their risk of resmoking.

Third, the overall program adherence rate was favorable at 70.7%, and the exclusive HTP users had a higher adherence rate than the exclusive cigarette users. Kato et al [17] previously reported that program adherence was 59.9% at week 24 in a study that also used the Ascure program [17]. Our study had a high proportion of male participants and exclusive HTP users, who tend to have a high adherence rate, likely contributing to the overall improvement in adherence [9]. Moreover, approximately half of current dual users in Japan use HTPs because these products might help them quit using cigarettes [21]. Further, exclusive HTP users are more likely to report that HTPs are helpful for smoking cessation than dual users [28]. Exclusive HTP users might have higher motivation to quit using tobacco and awareness of the importance of quitting than traditional smokers, which might have contributed to the high program adherence rate.

### Strengths and Limitations

The strengths of our study include analyzing data from a high number of participants: the exclusive HTP group included 1524 people. We also used salivary cotinine testing as a biochemical validation of the success of smoking cessation. Self-reports of smoking status can be inaccurate, and the salivary cotinine test is known to be a highly sensitive measure to ascertain smoking status [29]. Measuring nicotine metabolites in saliva is also useful in HTP users since HTPs, like conventional cigarettes, contain nicotine [30]. Despite these strengths, this study had some limitations. First, there was no control group, which makes it difficult to interpret the degree to which the app contributed to smoking cessation success. Second, we did not adjust for unmeasured confounding factors, such as sociodemographic factors (ie, occupation, education, and income), smartphone use in daily life, adverse events, or continued use of nicotine products. Further study is needed to more strictly assess the efficacy of the program with a control group, such as a group that uses a sham app, and with measurement of details of the above confounding factors. Third, we used the original nicotine dependence score of the NCDS for determining nicotine dependence at baseline. Although the NCDS was created based on the Tobacco Dependence Screener (TDS) and Fagerstrom Test for Nicotine Dependence (FTND) and has already been used in several of our studies, including clinical trials [14,18], using this scale might have affected the accuracy of our evaluation of nicotine dependence among the program participants, because the NCDA includes measurements of self-perceived smoking behavior. Fourth, we used CAR 21-24 results as the primary outcome. Although CAR 9-24 is important for the long-term assessment of smoking cessation [31], we could not collect data on the success or failure of smoking cessation from weeks 13 to 20 because of the optional counseling-session period. Finally, the true success or failure of smoking cessation is unknown for participants who dropped out of the program or voluntarily used nicotine products. We considered participants who dropped out or had false-positive results in saliva cotinine testing, even if they had truly quit smoking, as having failed smoking cessation. We might have thus underestimated the smoking cessation success rate in all 3 groups.

### Conclusion

We found that exclusive HTP users had higher CARs and adherence rates compared with exclusive cigarette users, indicating better results from the Ascure online smoking cessation program. This program might be a useful smoking cessation option for HTP users. Nevertheless, since smoking cessation is not easy for dual users, further research on the complex mechanisms of smoking cessation in dual users is warranted.

## Appendix

Multimedia Appendix 1 Supplementary methods. Explanation of the Nicotine Dependence Cognitive Scale (NDCS).

## Abbreviations

CAR: continuous abstinence rate
HTP: heated tobacco product
IPW: inverse probability weighting
NDCS: Nicotine Dependence Cognition Scale

## Data Availability

The data underlying this study have been prepared under ethical considerations for specific research purposes and are not available to the public.
