# Supplementary figures and images for: The Usefulness of a Smartphone App–Based Smoking Cessation Program for Conventional Cigarette Users, Heated Tobacco Product Users, and Dual Users: Retrospective Study
Source: J Med Internet Res. 2023 Mar 17;25:e42776. doi: 10.2196/42776 (PMC10131758; doi:10.2196/42776)

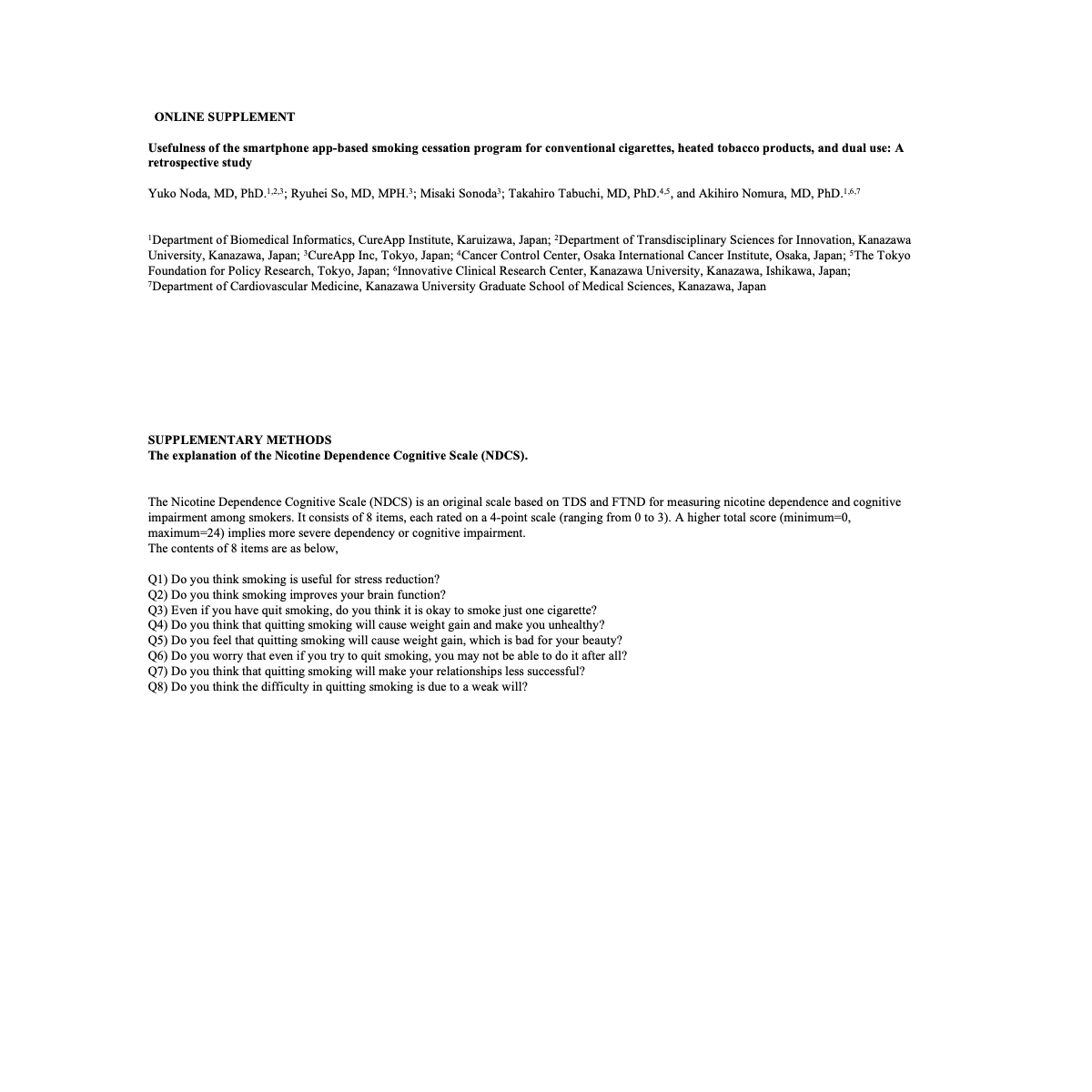

Supplement: Multimedia Appendix 1 [file jmir_v25i1e42776_app1.png]
